# The Importance of a PM&R Consultation for Becker Muscular Dystrophy Patients Admitted with Cardiomyopathy

**DOI:** 10.1155/2024/3145086

**Published:** 2024-03-22

**Authors:** Neyha Cherin, Shivani Patel, Michelle Jukic

**Affiliations:** Department of Physical Medicine and Rehabilitation, Penn State Health Milton S. Hershey Medical Center, Hershey, PA, USA

## Abstract

Becker muscular dystrophy (BMD) is a rare genetic disorder that is associated with significant cardiac compromise, including heart failure and cardiomyopathy. Given the significant cardiac impact of the disease, patients are commonly hospitalized under the care of cardiologists. While it is imperative to address the acute cardiac challenges these patients face, it is crucial to not disregard the musculoskeletal derangement that occurs from this underlying disease and how acute hospitalization can exacerbate these issues. While literature focuses heavily on providing management protocols to address these acute cardiac complications, it is also important for providers to feel supported in addressing the functional implications that hospitalized BMD patients may face. An early PM&R consultation in the inpatient setting can be useful in identifying and addressing the functional impairments and subsequent comorbidities of BMD patients. PM&R teams can provide oversight from multiple avenues including the psychosocial, neurocognitive, durable medical equipment, and pain management perspectives and assist with transition of care to the postacute rehabilitation setting. The ultimate goal of the PM&R provider is to work alongside the primary service and patient in order to assist with retaining independence, improving patient satisfaction, and most importantly improving quality of life both inside and outside of the hospital setting.

## 1. Introduction

There is no scientific literature demonstrating the importance of physical medicine and rehabilitation (PM&R) consultations for Becker muscular dystrophy (BMD) patients admitted to the hospital with cardiomyopathy. BMD is a rare, X-linked recessive condition, resulting from a mutation in the dystrophin gene [1]. Throughout general medical training, it is taught to monitor for progressive muscle weakness and skeletal muscle degeneration, given dystrophin's vital role in the stabilization of the muscle fiber cytoskeleton on the extracellular matrix. As specialists, however, cardiologists are acutely aware of the extensive cardiac manifestations of the disease. Cardiac tissue is a subtype of striated muscle [2]; therefore, it is no surprise that cardiomyopathy is a likely manifestation of those affected by BMD [3]. In fact, heart failure from dilated cardiomyopathy is the most common cause of death in BMD patients [4]. Scientific literature provides clinicians with guidance on the management of the cardiac needs of these patients in both the inpatient and outpatient settings. While it is imperative to treat the cardiac component of BMD, it is also crucial to not neglect the functional implications an acute hospitalization can have on those with preexisting functional deficits. When overlooked, this can compromise medical outcomes, lead to increased healthcare spending, and negatively impact the quality of life of BMD patients. We present a case of a 50-year-old-male with a history of BMD who required multiple prolonged hospitalizations in the span of four months from worsening heart failure and cardiomyopathy, which resulted in his drastically altered ability to remain independent posthospital discharge. Prior to the hospitalizations, the patient lived and functioned independently. Unfortunately, as his functional needs were not addressed throughout his hospital encounters, he was unable to return home due to his new functional and activities of daily living (ADL) support needs. The patient subsequently required moving in with family across state lines. We hypothesize that if PM&R consultations were obtained throughout his hospital stays, the PM&R team could have utilized a multimodal treatment approach throughout each admission, focusing on obtaining appropriate therapy services, durable medical equipment, and psychosocial support services, as well as providing assistance in transitioning to the outpatient realm with the ultimate goal of functional restoration, maintenance of independence, and improvement in quality of life.

## 2. Case Presentation

A 50-year-old male with a history of Becker muscular dystrophy (BMD) complicated by cardiac involvement including heart failure (HF), cardiomyopathy, and paroxysmal ventricular tachycardia (VT) presented to the emergency department (ED) on three separate occasions from August to November with a complaint of chest discomfort after several episodes of his dual-chamber implantable cardioverter-defibrillator (ICD) firing. Of note, the patient was diagnosed with BMD at the age of 43 after new seizure activity and new cardiomyopathy diagnoses, which prompted extensive genetic and neurologic workup. Upon his BMD diagnosis, the patient lived independently in a stair accessible apartment, worked full time in construction, and appropriately had a multidisciplinary network of clinicians supporting his multiorgan needs including primary care, cardiology, neurology, PM&R, and therapy services. Shortly after his BMD and cardiomyopathy diagnoses, the patient was placed on disability for his new cardiac needs (cardiomyopathy requiring ICD placement, VT requiring ablation, and HF with reduced ejection fraction, as seen in Tables 1–3). The patient continued to successfully complete his ADLs and ambulate with a cane and did not require any additional home services to live independently.

At the age of 50, the patient presented to the ED after his ICD continued to fire over a three-month time frame. During the first two hospitalizations, the patient underwent extensive cardiac workup for management of uncontrolled VT. Throughout the encounters, a PM&R consultation was not obtained, and the patient was discharged home after the stabilization of his acute cardiac issues. After the first encounter, while the patient continued to live independently, he now required a rollator due to a decline in mobility. After the second encounter, the patient was no longer able to complete stairs and required moving in with his family across state lines for more assistance with ADLs and mobility.

Shortly after moving, the patient continued to experience ICD firing, prompting another hospital visit, this time at a center with PM&R services. The patient was admitted to the cardiology service, and his cardiac needs were promptly addressed. The patient required treatment for ventricular tachycardia storm and underwent cardioversion and ablation. Guideline-directed medical therapy was initiated, and he was able to be weaned off of amiodarone, lidocaine, and esmolol infusions, with transition to oral medications for heart rate control. One week following ablation, postop device interrogation revealed no evidence of recurrent arrhythmias. Towards the end of his stay, a PM&R consultation was requested to assist with disposition needs. Upon meeting the patient, the PM&R team was not only able to assist with postacute transition of care as requested but also to facilitate oversight in the unique, multimodal functional needs of the patient. An individualized treatment plan was curated to optimize the patient's function, including physical exercise recommendations to maintain a safe balance between muscle atrophy and overuse, custom ankle foot orthosis bracing for his bilateral foot drop, wheelchair ordering for his new impaired mobility, assistance with his sleep-wake cycle, and medical oversight of his anxiety and depression needs. Unfortunately, given that roughly four months had lapsed since his functional needs were first addressed, the patient experienced a significant decline in function by the time of PM&R engagement and therefore required discharge to an inpatient rehabilitation setting to optimize his functional recovery and ADL performance. Fortunately for this patient, he had strong family support, with family quickly taking on caregiver responsibilities following inpatient rehabilitation discharge. The PM&R consult service also followed the patient as he transitioned from the acute hospital to inpatient rehab and assisted with engaging outpatient rehabilitation services to ensure ongoing functional oversight and patient and caregiver support.

## 3. Discussion

BMD is a very rare, X-linked recessive inherited disorder resulting in progressive muscle weakness. Dystrophin is a gene which is located throughout the body. When mutated, as in BMD, it results not only in muscular impairment but also in significant cardiac compromise [1, 2]. The importance of early cardiology involvement after BMD diagnosis is well known, given that heart failure from dilated cardiomyopathy is its leading cause of death [4]. There is significant literature guiding cardiologists on the cardiac management of BMD patients in both inpatient and outpatient settings. In each healthcare setting, cardiologists are evaluated not only on their ability to initiate appropriate treatment management protocol but also on patient satisfaction levels. It would be remiss for cardiologists to ignore that while cardiac medical treatment may be of utmost importance, patient satisfaction can be impacted by lack of functional management, which may alter patient perception of the quality of medical treatment received. To ensure both areas are sufficiently addressed, it is important for cardiologists to recognize how functional compromise can drastically impact patient satisfaction and quality of life. PM&R consulting providers can assist cardiologists with this comprehensive approach. To the author's knowledge, there is no scientific literature addressing the importance of a PM&R consultation on this patient population in the acute care setting.

To optimize care, it is important to address both the cardiac and functional needs of BMD patients. Given the rare nature of the disease, with a prevalence of 1.53-3.6/100,000 males worldwide [5, 6], and the variability in age of onset, symptom presentation, and multiorgan involvement, this level of patient complexity can historically make BMD a difficult diagnosis to treat. Additionally, given that the average life expectancy for BMD ranges from about 4 to 5 decades, it is virtually guaranteed that patients will need extensive multidimensional support as they age. It therefore is no surprise that an interdisciplinary team management approach should be utilized whether it is in the outpatient or inpatient setting. BMD patients require close functional monitoring in addition to addressing the specific organ complaint which brings them to the hospital. From an outpatient perspective, consensus guidelines support a multidisciplinary treatment approach, including support for the cardiovascular, pulmonary, integumentary, neuropsychological, and muscular systems [7]. Rehabilitation management is a key component in this interdisciplinary network to ensure successful, concomitant management of both medical and functional care [8]. This outpatient formula ensures a well-balanced approach and should be carried over in an inpatient setting as well. The absence of a comprehensive treatment approach, including early rehabilitative care, can severely impact functional outcomes and result in a significant decline in quality of life, as seen with this patient. Interestingly, the standard of care for patients with Duchenne muscular dystrophy (DMD), which is a more severe and common form of muscular dystrophy due to the severe absence of dystrophin, has been discussed more heavily in literature despite the similar etiology and presenting signs and symptoms to BMD. The DMD care consideration model was developed to outline the comprehensive and multidisciplinary care of this disease with the goal of improving patient care and standardizing care. The model highlights different stages of management in various domains, including neuromuscular, rehabilitation, endocrine, gastrointestinal and nutritional, respiratory, cardiac, bone health, orthopedic, psychological, and transition management [9]. Although BMD is less severe and frequent, it shares many characteristics with DMD. Therefore, a similar model to guide treatment and management would help standardize care and improve outcomes in those who are suffering from this disorder. We present the case of a 50-year-old BMD patient with a drastic decline in his ability to ambulate and perform ADLs in the setting of multiple hospitalizations and limited PM&R multidisciplinary support. Given that the patient was no longer able to independently ambulate or care for himself, he required a change in living situation, moving in with family across state lines. We hypothesize that if the patient received PM&R support throughout the continuum of his hospitalizations, his rate of functional decline could have been mitigated, allowing him the opportunity to continue living independently with appropriate community supports in place.

It is well known that hospitalizations have negative effects on muscular strength and endurance resulting in a functional decline in the general patient population [10]. Bed rest results in impaired organ function throughout the body including the musculoskeletal, cardiovascular, respiratory, psychological, cognitive, and integumentary systems [11]. It is no surprise that these effects are amplified in patients with a history of preexisting muscular dysfunction, as seen in patients with BMD. Unfortunately, if not addressed, the resulting impaired muscle stability can irreversibly impact BMD patients' quality of life [12]. While literature does support early bed mobility and therapy services throughout hospitalization for the general population, there is controversy regarding the appropriateness of physical exercise and level of intensity of muscle engagement for those with BMD. Historically, it has been taught that physical exercise should be discouraged in the BMD population due to the underlying belief that the muscles of those with BMD do not have the ability for regeneration postexercise, and therefore, physical exercise should be discouraged within the treatment plan [13]. In this patient's case, we hypothesize that this viewpoint led to virtually no therapy service engagement throughout his three hospitalizations. Therapy was only utilized one time at the end of the third hospitalization to assess if home discharge was a safe option. As the literature on muscular dystrophy has grown, this view that physical exercise should be avoided in BMD patients is no longer in favor. With the support of scientific data, census guidelines recommend monitored physical exercise to prevent contracture formation, improve range of motion, and maintain independence [13]. Bostock et al. studied the effects of resistance exercise training on strength and functional ability in those with BMD and found support for the use of resistance training in the treatment program [14]. It is important, however, to ensure that physical exercise is appropriately monitored, as muscle metabolism remains variable amongst BMD patients, which can subsequently negatively impact the cardiovascular and pulmonary systems if not observed closely. In this patient, physical exercise and reinforcement of the importance of getting out of bed to chair daily could have improved endurance, stability, muscle strengthening, and mood. However, given his cardiac complexity, it is no surprise that there was hesitation to institute this plan. As specialists in neuromuscular medicine, this physical exercise and mobility regimen could have been overseen and adjusted appropriately by the PM&R team to ensure ongoing cardiac and pulmonary stabilization, while also focusing on the goal of minimizing both disuse atrophy and functional decline.

Not only can PM&R consultants assist with exercise recommendations during the acute hospitalization, but they can also aide the cardiology service in overseeing various other aspects of care that arise from other underlying comorbidities in patients with BMD. This can include oversight from a psychosocial, neurocognitive, durable medical equipment, and pain management perspective to name a few. In this patient's case, if PM&R services had been obtained earlier during the hospitalization, durable medical equipment needs could have been identified sooner. This includes nightly resting hand splints to decrease wrist and finger flexion contracture formation and improve passive range of motion, as well as bilateral ankle foot orthoses to assist with foot drop allowing for safe mobilization, prevention of ankle plantarflexion contracture, and optimization of participation in therapies. Without these adjunct treatments, disuse atrophy and decreased mobilization increase the risk of contracture formation and ultimately impede ambulatory ability and community independence, as seen in our patient's case.

While this patient's cardiac care was properly addressed during each encounter, by the time PM&R was involved in his case, it was apparent that he needed psychosocial support. Mood disorders, in the form of anxiety and depression, are common in BMD, given that these patients deal with lifelong progressive functional decline and the constant need for ongoing adjustments at each stage of disease progression [15]. Additionally, Mori-Yoshimura et al. found that BMD diagnosis itself is a risk factor for psychiatric diseases, given that patients face daily functional stressors. It is, therefore, not surprising that our patient had a history of underlying anxiety and depression and, with the stressors of his three recent hospitalizations, an acute flare in mood symptomatology. PM&R providers can provide medical and psychosocial support for these patients during their hospitalization and beyond.

Pain is a common manifestation of those with BMD, with studies noting that roughly 80% of patients experiencing pain at least once weekly [16]. The etiology can be multifaceted, ranging from contractures and fractures to muscle fatigue and cramping [17]. Given the multidimensional source and impact of BMD pain, it is noted to be under-recognized and inadequately treated by clinicians [16]. Throughout training, PM&R providers are taught the importance of pain management and its impact on functional recovery. With the integration of PM&R services into the patient's care plan, PM&R providers can assist with identifying the etiology of pain and subsequently implementing appropriate treatment regimens including oral, topical, psychological, and injection management if necessary.

As in this patient's case, PM&R was eventually engaged appropriately to assist with transition to postacute rehabilitation services. However, if the service had been engaged earlier, PM&R could have worked alongside the patient, caregiver, and social work team to assist with earlier transition to the community. This could be implemented specifically by obtaining inpatient and outpatient rehabilitation services, including both physician and therapy services, as well as home nursing services, given his new functional decline. In addition, the process for a custom wheelchair evaluation for his worsening ambulatory dysfunction could have been initiated. Fortunately, for this patient, the close support of his family allowed him to remain in the community once discharged from rehab. Unfortunately, not all patients have that level of support and can subsequently require placement in a skilled nursing facility once the functional decline has reached the point that they can no longer live independently.

As cardiologists in the hospital setting, the focus of care is rightfully on the acute cardiac needs of those with BMD. It is crucial, however, to also approach these patients globally in order to ensure that all of their functional requirements are addressed. While the acute medical issue may be appropriately treated and the patient medically stabilized, other outcomes including patient satisfaction, length of stay, and quality of life can suffer if the underlying functional barriers are not addressed promptly. By engaging an interdisciplinary care team, including PM&R services, during the early stages of hospitalization, there will likely be a decrease in unnecessary healthcare spending long term, a decrease in hospital length of stay by initiating the process of determining the next level of rehabilitation (or postacute rehabilitation needs) sooner, and most importantly an improvement in patient satisfaction and quality of life by maximizing the likelihood of maintaining independence in the community.

## Figures and Tables

**Table 1:** Echocardiogram revealing heart failure with reduced ejection fraction.

Summary
1. Dilated left ventricle with moderate-severely reduced systolic function, EF 36% by Simpson's biplane.
2. Global hypokinesis with inferior and inferolateral akinesis.
3. Eccentric hypertrophy is present.
4. Restricted posterior mitral valve leaflet with mild-moderate regurgitation.
5. Severely dilated left atrium size.
6. Normal estimated pulmonary artery pressures, estimated PASP 29 mmHg.
7. Normal size RV with mildly reduced systolic function.
8. Normal IVC size and inspiratory collapse.
9. Compared to the most recent previous echocardiogram, the LV appears slightly less vigorous on the present study. No other major changes.

EF: ejection fraction; IVC: inferior vena cava; LV: left ventricle; PASP: pulmonary artery systolic pressure; RV: right ventricle.

**Table 2:** Echocardiogram revealing increased left atrium volume.

Atria		
Name	Value	Normal
LA dimensions		
LA area (4C)	24.3 cm^2^	
LA length (4C)	6.1 cm	
LA area (2C)	26.9 cm^2^	
LA length (2C)	5.5 cm	
LA volume (4C A-L)	81.77 ml	
LA volume (2C A-L)	111.70 ml	
LA volume (BP A-L)	101 ml	18-58
LA volume index (BP A-L)	52.14 ml/m^2^	≤34.00
RA dimensions		
RA area (4C)	16.5 cm^2^	≤18.0

LA: left atrium; RA: right atrium; cm: centimeters; ml: milliliters.

**Table 3:** Echocardiogram revealing increased left ventricle mass.

Ventricles		
Name	Value	Normal
LV dimensions 2D/MM		
IVS diastolic thickness (2D)	0.8 cm	0.6-1.0
LVID diastole (2D)	7.3 cm	3.6-5.6
LVIW diastolic thickness (2D)	0.7 cm	0.6-1.0
LVID systole (2D)	6.3 cm	2.5-4.0
LV mass (2D cubed)	238.70 g	88.00-224.00
LV mass index (2D cubed)	0.01 g/cm^2^	0.00-0.01
Relative wall thickness (2D)	0.18	

LV: left ventricle; IVS: interventricular septum; LVID: left ventricle inner dimension: LVIW: left ventricle inner wall; cm: centimeters.

## Data Availability

The literature review data supporting this case report are from previously reported studies and datasets, which have been cited. The processed data are cited at relevant places within the text as references #1-17.
